# Peroxisome proliferator-activated receptor alpha agonist suppresses neovascularization by reducing both vascular endothelial growth factor and angiopoietin-2 in corneal alkali burn

**DOI:** 10.1038/s41598-017-18113-3

**Published:** 2017-12-19

**Authors:** Takeshi Arima, Masaaki Uchiyama, Yuichiro Nakano, Shinya Nagasaka, Dedong Kang, Akira Shimizu, Hiroshi Takahashi

**Affiliations:** 1 Department of Ophthalmology, Nippon Medical School, Tokyo, Japan; 2 Department of Analytic Human Pathology, Nippon Medical School, Tokyo, Japan

## Abstract

We investigated the effect of a peroxisome proliferator-activated receptor alpha (PPARα) agonist ophthalmic solution in wound healing using a rat corneal alkali burn model. After instillation of a selective agonist of PPARα, fenofibrate, onto the burned cornea, PPARα-positive cells were observed in vascular endothelial cells, and there was upregulation of mRNA of PPARα in corneal stroma. Fenofibrate suppressed expression of neutrophils and macrophages during the early phase, and development of neovascularization and myofibroblast generation during the late phase. Fenofibrate reduced not only mRNA expression of vascular endothelial growth factor-A but also angiopoietin-1 and angiopoietin-2. Furthermore, fenofibrate suppressed scar formation by reducing type III collagen expression. These data suggest that a PPARα agonist ophthalmic solution might be a new strategy for treating corneal wounds through not only anti-inflammatory effects but also by preventing neovascularization.

## Introduction

A transparent cornea is essential for good vision. Suppression of scar tissue formation during cornea wound healing is key to maintaining corneal transparency. Since inflammation and neovascularization are known to be deeply involved in corneal scarring, agents that can suppress these phenomena have long been sought. One of the new suppression candidates is peroxisome proliferator-activated receptors (PPARs). PPARs are nuclear hormone receptors that belong to the steroid hormone receptor superfamily^1,2^. PPARs are important factors in adipocyte differentiation and lipid metabolism^3^. There are three isoforms of PPARs: PPAR alpha (α), PPAR delta (δ), and PPAR gamma (γ).

Recent studies have shown that PPARs have roles not only in lipid metabolism but also in inflammation^4,5^. Several studies have reported finding that PPARα agonists have protective and anti-inflammatory roles in retina^6–9^. Use of an *in vitro* corneal model has revealed that anti-inflammatory effects of PPARα occurred in conjunction with reduced interleukin (IL) -6 and IL-8 expression^10^. Anti-inflammatory effects for PPARδ have been reported also during corneal epithelial wound healing^11^. Furthermore, we previously found anti-inflammatory effects for PPARγ in the rat corneal alkali burn model^12^.

On the other hand, PPARα has been reported to exhibit specific proinflammatory effects in the absence of lipopolysaccharide^13^. Another report has shown additionally the presence of tubular damages in the kidney as a consequence of excessive serum accumulation of a PPARα agonist^14^. Since the role of PPARα in inflammation appears to be dependent on the specific situation, there has yet to be a detailed investigation of corneal wound healing. In addition, PPARα has been reported also to be present in vascular endothelial cells^15,16^, suggesting its involvement in the neovascularization process.

In our present study, after compounding an ophthalmic solution of fenofibrate, which is a selective agonist of PPARα, we investigated anti-inflammatory and anti-neovascularization effects of the solution in a rat alkali burn model. We found suppressive effects of PPARα in inflammation, fibrosis formation, and neovascularization in alkali burned cornea. Interestingly, anti-neovascularization effects of PPARα involved downregulation not only of vascular endothelial growth factor (VEGF) -A, but also angiopoietin (Ang) expression.

## Results

### Wound healing after alkali burn

**Figure 1:**
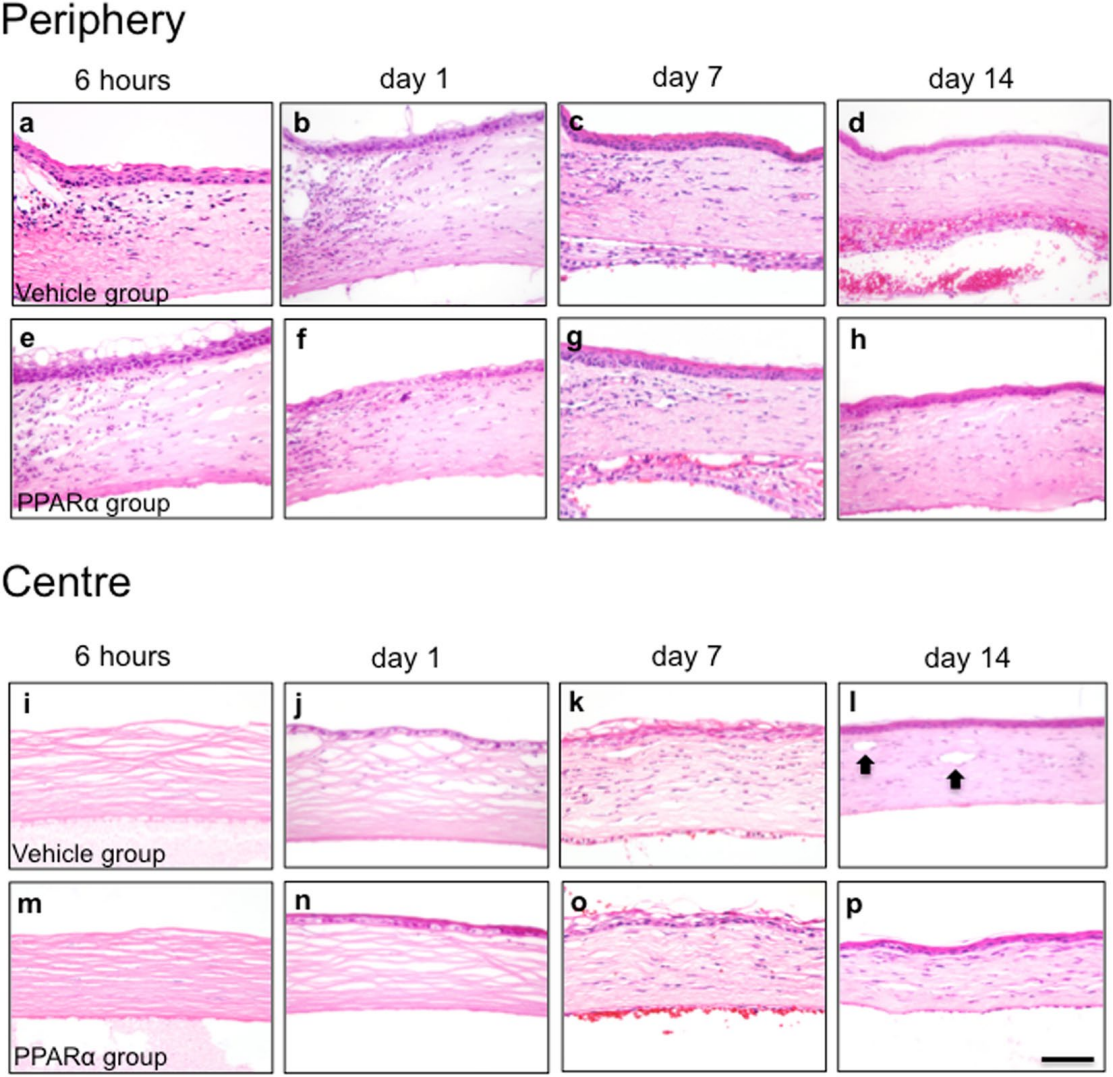
Wound healing after alkali burn. Development of corneal wound healing after alkali burn injury in vehicle (**a–d**: periphery, **i**–**l**: center) and PPARα (**e**–**h**: periphery, **m**–**p**: center) groups. Various inflammatory cells occurred in peripheral corneal regions within 24 hours, and infiltrated to centre of cornea by day 7. During late phase, neovascularization (black arrows; **l**) was observed in central stroma at day 14. Bar, 50 μm.

Effects of PPARα ophthalmic solution were investigated by performing histological analysis using hematoxylin and eosin (HE) staining. At 6 hours and at day 1 (early phase) after alkali burn, there was an increased infiltration of various inflammatory cells in corneal limbus (Fig. 1a,b,e and f). At 6 hours after injury, we noted peeling of corneal epithelium and oedema of the stroma in the centre of the cornea (Fig. 1i and m). On day 1, however, epithelial cells were already regenerating (Fig. 1j and n). Inflammatory cells observed at corneal limbus during the early phase were found to be infiltrating the corneal centre on day 7 (Fig. 1k and o). By day 14, we noted neovascularization at the corneal centre (Fig. 1l). PPARα group exhibited a lesser degree of inflammatory cell infiltration and neovascularization as compared to vehicle group.

### Anti-inflammatory roles of PPARα agonist ophthalmic solution

**Figure 2:**
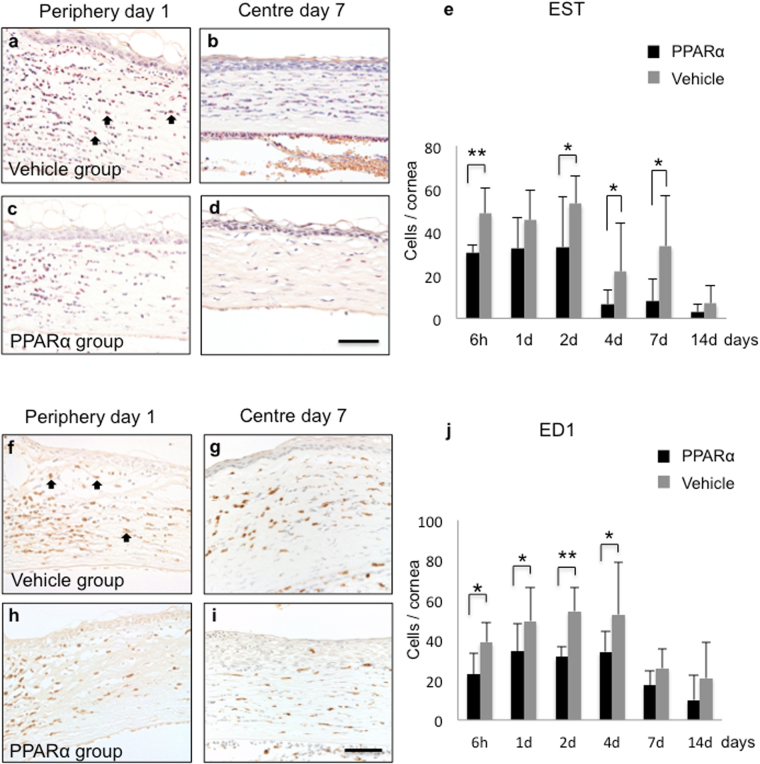
Anti-inflammatory roles of ophthalmic solution of PPARα agonist. (**a**–**d**) EST staining was used to evaluate neurophil infiltration into burned stroma (black arrows; (**a**) PPARα treatment reduces number of neutrophils in peripheral stroma at day 1 (**c**) and in central stroma at day 7 (**d**) as compared with vehicle group (**a,b**). Bar, 50 μm. (**e**) Bar chart of number of EST-positive cells shows a statistically significant difference between PPARα (black bars) and vehicle (gray bars) groups. ** Or * indicates significance at *P* < *0.01* or *P* < *0.05*. (**f**–**i**) ED1 staining was used to evaluate infiltration by macrophages (black arrows; **f**). PPARα treatment reduces number of macrophages (**h,i**) as compared with vehicle group (**f,g**). Bar, 50 μm. (**j**) Bar chart of number of ED1-positive cells shows a significant difference in number of macrophages between PPARα (black bars) and vehicle (gray bars) groups. ** Or * indicates significance at *P* < *0.01* or *P* < *0.05*.

To investigate anti-inflammatory effects of PPARα ophthalmic solution, Naphtol AS-D chloroacetate esterase (EST) staining and immunohistochemical analysis of CD68 antibody (ED1) were performed. In both groups, EST-positive neutrophils (Fig. 2a and c) and ED1-positive macrophages (Fig. 2f and h) were noted in corneal limbus at day 1. By day 7, these inflammatory cells were infiltrating the corneal centre (Fig. 2b,d,g and i). Numbers of neutrophils (Fig. 2e) and macrophages (Fig. 2j) were significantly lower in PPARα group versus vehicle group during the early phase.

### Effect of PPARα agonist on nuclear factor-kappa B (NF-κB)

**Figure 3:**
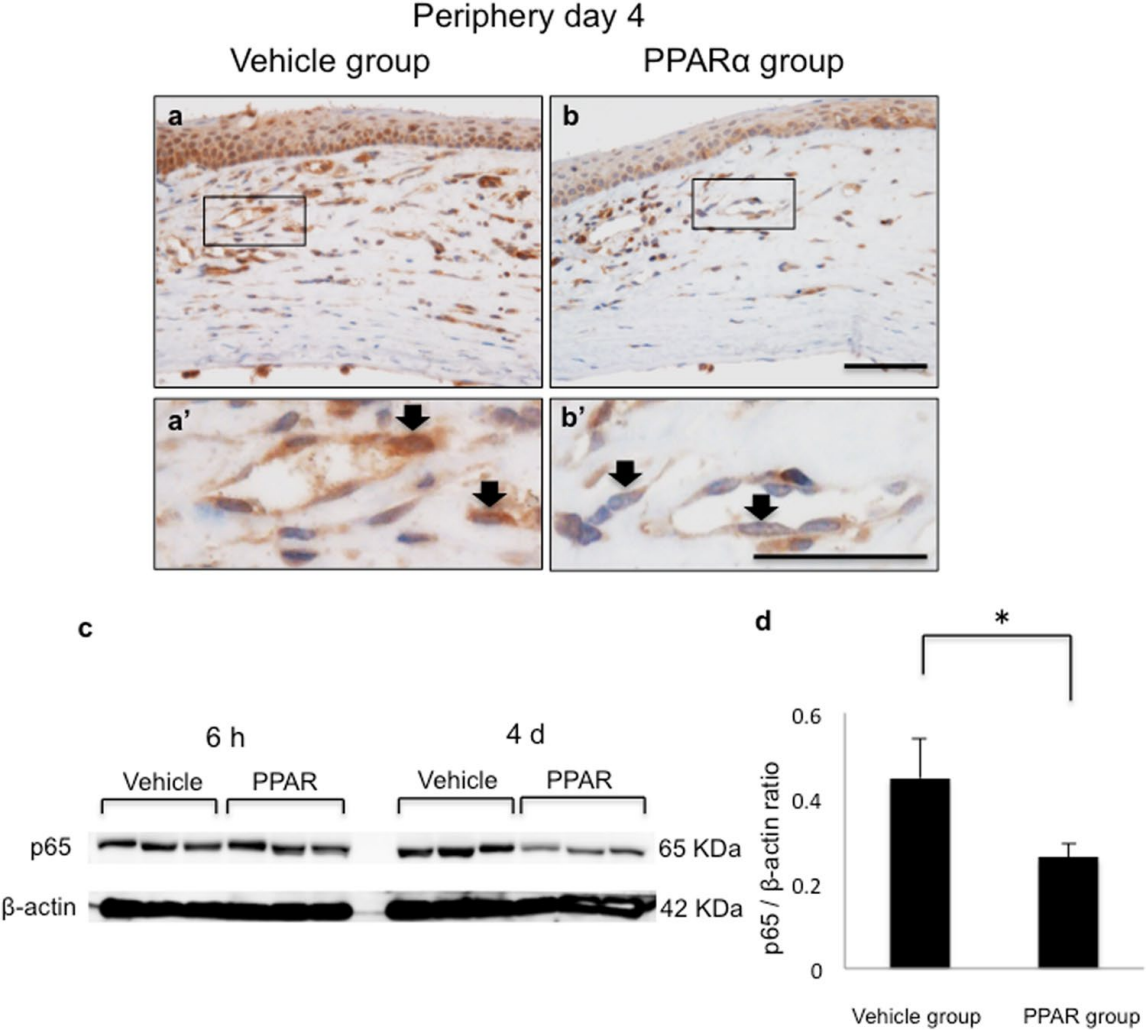
Expression levels of NF-kB and protein levels of NF-kB in both groups. (**a,b,a’** and **b’**) p65 immunostaining on day 4 was performed to examine production of NF-kB. PPARα group had lower expression levels of NF-kB compared with vehicle group. Frames **a’** and **b’** show higher magnification pictures of boxed areas in frames **a** and **b**. Vehicle group exhibited strong staining in nucleus of inflammatory cells and vascular endothelial cells compared with PPARα group (black arrows; **a’,b’**). Bar, 50 μm (**a,b**); 20 μm (**a’**,**b’**). (**c**) p65 protein levels in corneas from 6 hours to day 4 after injury was investigated using western blotting. (**d**) Amounts of each protein on day 4 were quantified with results and expressed as ratios to β-actin (control) protein amounts. The PPARα group had significantly less expression of NF-kB compared with the vehicle group. *Indicates significance at *P* < *0.05*.

To examine PPARα effects on NF-kB expression, immunohistochemical analysis and western blotting were performed using anti-p65 antibody. In inflammatory cells and vascular endothelial cells after alkali injury, NF-kB expression was localized in the nuclear area in vehicle group, while it was expressed in cytoplasm in PPARα group (Fig. 3a,b,a’ and b’). Western blotting revealed there was less expression of NF-kB in the PPAR versus vehicle group (Fig. 3c and d).

### Expression of PPARα in rat cornea

**Figure 4:**
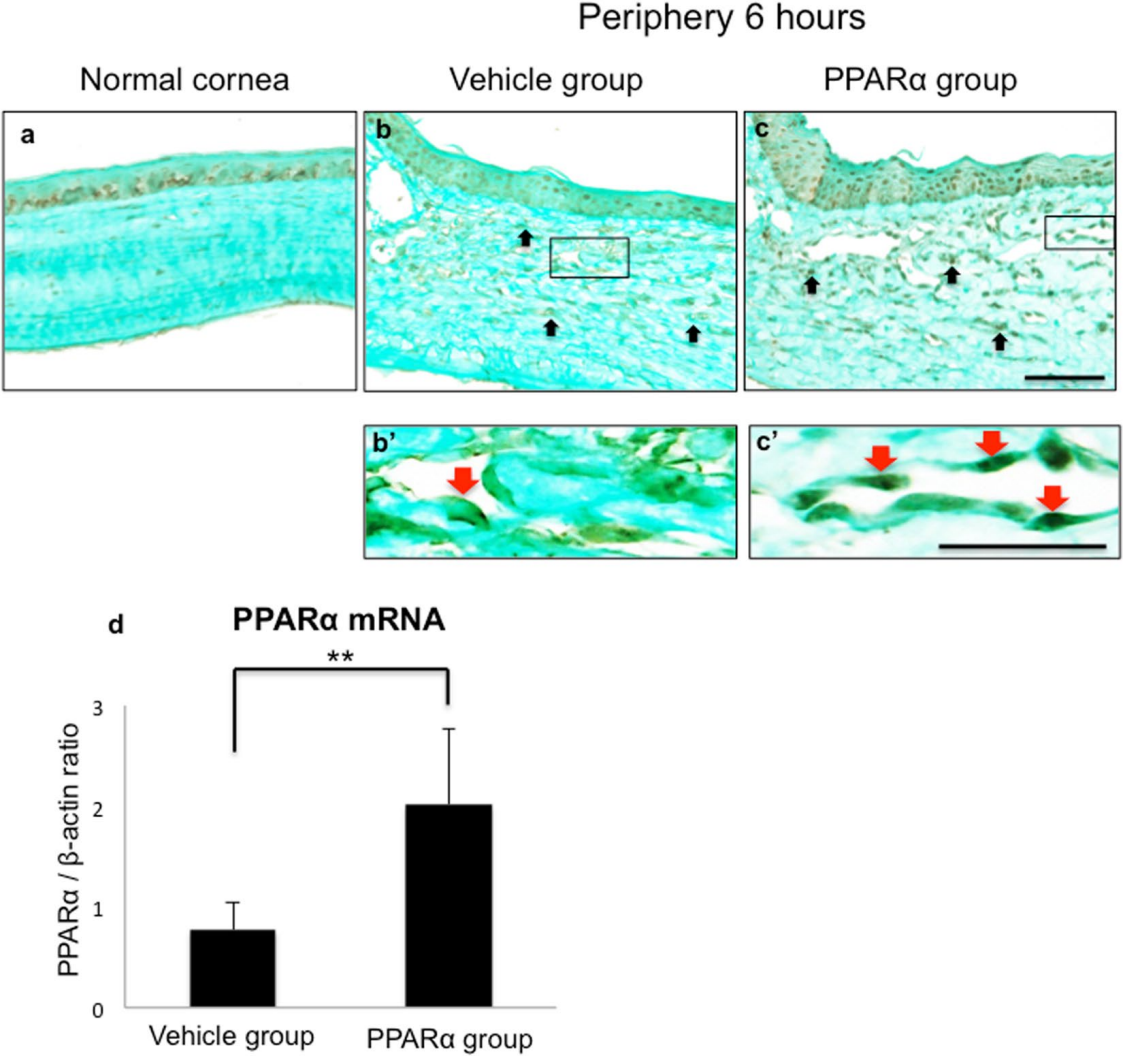
Expression of PPARα in rat cornea. (**a**–**c, b’,c’**) PPARα localized in normal corneal epithelial basement area (**a**) and in inflammatory cells in stroma at 6 hours (black arrows; **b,c**). Frames **b’** and **c’** show higher magnification pictures of boxed areas in frames **b** and **c**. PPARα-positive cells in PPARα group were more prominent and stained strongly in nucleus of vascular endothelial cells (red arrows; **b’,c’**). Bar, 50 μm (**a–c**); 20 μm (**b’,c’**). (**d**) Real-time RT-PCR showed marked upregulation of mRNA expression of PPARα in burned cornea after PPARα treatment as compared with vehicle group at 6 hours. **Indicates significance at *P* < 0.01.

After instilling fenofibrate, PPARα staining was performed in order to investigate changes in expression of PPARα in burned cornea after 6 hours. PPARα-positive cells were observed in epithelial basement cells of normal rat cornea (Fig. 4a). After alkali injury, PPARα-positive cells were observed in inflammatory cells in both groups (Fig. 4b and c). Compared to vehicle group, PPARα group had prominent PPARα-positive cells that exhibited intense intranuclear staining in vascular endothelial cells (Fig. 4b’ and c’). Use of real-time reverse transcription polymerase chain reaction (RT-PCR) to determine mRNA expression of PPARα indicated that instillation of PPARα agonist increased expression of mRNA of PPARα in cornea (Fig. 4d).

### Suppression of neovascularization by PPARα agonist

**Figure 5:**
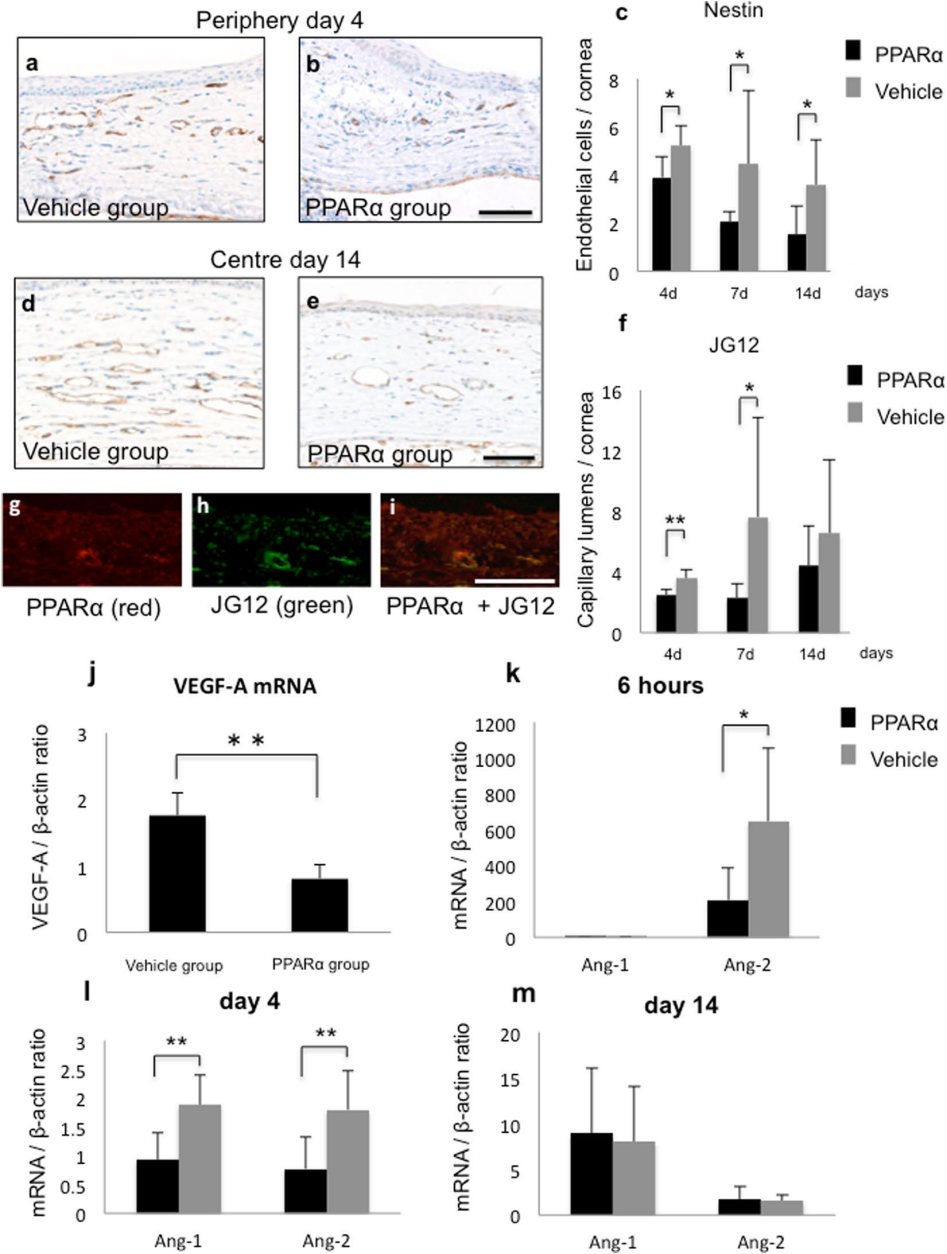
Suppression of neovascularization by fenofibrate. (**a–c**) Nestin stained endothelial cells were observed during early phase in peripheral stroma (**a,b**). Bar, 50 μm. Bar charts of number of nestin-positive cells (**c**) indicates that PPARα group (black bars) had a smaller number of newly formed endothelial cells compared to vehicle group (gray bars). *Indicates significance at *P* < *0.05*. (**d**–**f**) JG12-stained capillary lumens were observed during late phase in central stroma (**d,e**). Bar, 50 μm. Bar chart of number of JG12-positive cells (**f**) shows a statistically significant difference in number of JG12-stained capillary lumens between PPARα (black bars) and vehicle (gray bars) groups. ** Or * indicates significance at *P* < 0.01 or *P* < *0.05*. (**g**–**i**) Double immunofluorescence studies with PPARα (**g**) and JG12 (**h**) in burned corneal stroma at day 4 showed that PPARα was expressed in vascular endothelial cells (**i**). Bar, 50 μm. (**j**) Real-time RT-PCR showed marked suppression of mRNA of VEGF in burned cornea by PPARα group as compared with vehicle group at day 4. **Indicates significance at *P* < *0.01*. (**k**–**m**) Bar chart of mRNA expression of Ang-1 and Ang-2 at 6 hours (**k**), day 4 (**l**), and day 14 (**m**) showed that PPARα group (black bars) had low expression levels of Ang-1 and Ang-2 as compared with vehicle group (gray bars) during early phase. ** Or * indicates significance at *P* < *0.01* or *P* < 0.05.

To investigate neovascularization, nestin and aminopeptidase P (JG12) were immunostained. In both groups, nestin-positive endothelial cells (Fig. 5a and b) were observed in corneal limbus starting from day 4, with PPARα agonist significantly reducing nestin-positive endothelial cells (Fig. 5c). Subsequently, JG12-stained capillary lumens were noted in the corneal centre on day 14 (Fig. 5d and e). There was a significantly smaller number of capillary lumens in the PPARα group versus the vehicle group (Fig. 5f). Double immunofluorescence studies demonstrated that PPARα was expressed on vascular endothelial cells, suggesting that expression of PPARα was associated with neovascularization (Fig. 5g–i). There was lower mRNA expression of VEGF-A (Fig. 5j) in the PPARα versus vehicle group. Furthermore, mRNA expressions of Ang-1 and Ang-2 were significantly lower at 6 hours (Fig. 5k) and at day 4 (Fig. 5l). These results indicated that PPARα agonist ophthalmic solution prevented development and migration of neovascularization.

### Regeneration of corneal stroma and corneal transparency

**Figure 6:**
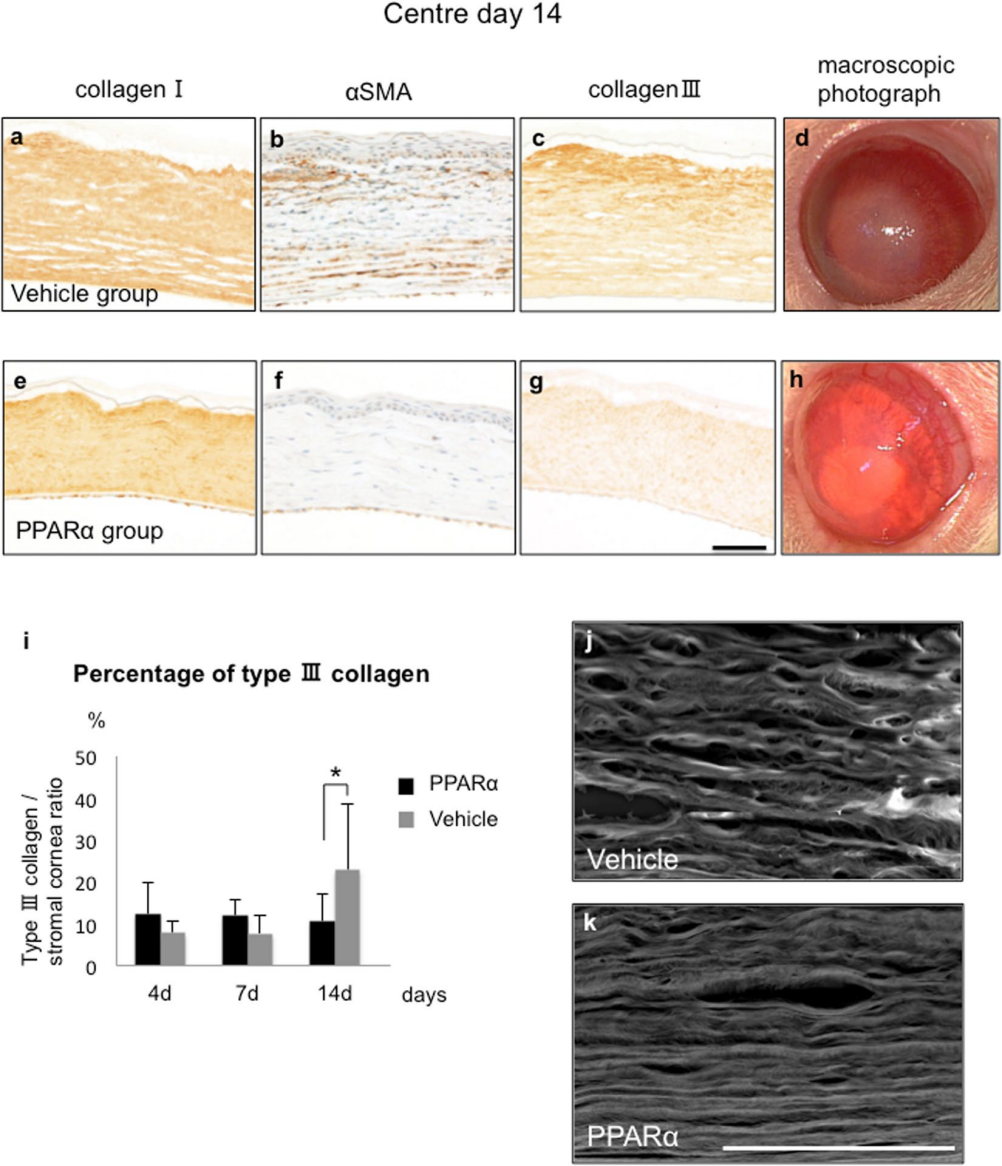
Regeneration of corneal stroma and corneal transparency. (**a**–**h**) Corneal stroma mainly consists of type I collagen (**a,e**). Although type III collagen and α-SMA were observed at injured area during healing process, PPARα group (**f,g**) exhibited less expression of type III collagen and α-SMA compared to vehicle group (**b,c**). Macroscopic observation showed ocular surface after fibrotic reaction in PPARα (**h**) and vehicle (**d**) groups. Bar, 50 μm (**a**–**c**,**e**–**g**). (**i**) Bar chart of percentages of expression rate of type III collagen/corneal stroma during late phase showed that PPARα group (black bars) had significantly lower percentages of type III collagen compared to vehicle group (gray bars). *Indicates significance at *P* < 0.05. (**j,k**) Comparison of collagen fibers by LV-SEM shows that collagen in PPARα group (**k**) was accompanied by a smooth surface as compared to vehicle group (**j**), during which, sequence was disturbed, and oedema was observed between fibers. Bar, 30 μm.

To observe fibrotic changes in corneal stroma during wound healing, we focused on alpha-smooth muscle actin (α-SMA) -positive myofibroblasts and type III collagen. Corneal stroma mainly consists of type I collagen (Fig. 6a and e). On day 14 after injury, we noted accumulation of α-SMA-positive myofibroblasts (Fig. 6b) and deposition of type III collagen (Fig. 6c). However, PPARα group exhibited a lower degree of α-SMA and type III collagen (Fig. 6f and g). Macroscopic photographs also showed that PPARα group had a higher corneal transparency and a lower central opacity compared to vehicle group (Fig. 6d and h). Percentages of type III collagen in corneal regions were significantly lower in PPARα group versus vehicle group (Fig. 6i). While there was a gradual increase in type III collagen area in vehicle group, lower percentages of type III collagen area were found in PPARα group. Low-vacuum scanning electron microscopy (LV-SEM) analysis showed the detailed sequence of collagens (Fig. 6j and k). Although PPARα group exhibited more regulated collagens and less corneal oedema compared to vehicle group, collagens observed in vehicle groups exhibited a wavy-like arrangement.

## Discussion

Alkali burn is one of the most severe corneal injuries, as it not only damages corneal epithelium and stroma but also causes acute inflammation that can lead to neovascularization^17,18^. Thus, alkali burn model is a suitable tool for investigating PPARα agonist effects on corneal injury. Our current study demonstrated suppressive effects of PPARα agonist on inflammatory cell infiltration, neovascularization, and scar formation in alkali-injured cornea. We also found that anti-inflammation and anti-neovascularization effects of PPARα were closely related pathologically.

With our present model, we first observed corneal infiltration of cells after injury, with PPARα group leading to a decrease in infiltration of approximately 30% during the early phase. Anti-inflammatory effects of this agonist have been reported to be caused by interference of PPARα on the activity of many proinflammatory transcription factors, such as signal transducer and activator of transcription (STAT), activator protein-1 (AP-1), and NF-kB pathway^19–22^. Results of immunohistochemistry and western blotting examinations suggested that PPARα agonist effects occurred through NF-kB pathway by reducing its expression. Expression of other transcription factors including c-Jun, c-Fos, STAT5b, and phosphorylated STAT (P-STAT) showed no significant difference between PPARα and vehicle groups (Supplementary Fig. S1). These agonist effects were confirmed by our findings that showed PPARα suppressed infiltration of inflammatory cells. Our results also indicated that there was a difference in both rate and order of infiltrations in accordance with type of inflammatory cells. We found that neutrophils infiltrated first followed by macrophages, with infiltrating neutrophil numbers larger than that observed for macrophages. These results were in agreement with previous study findings^12,23^. In addition, fenofibrate suppressed infiltrations of neutrophils and macrophages, regardless of phase.

Subsequently, we observed alterations in localization of PPARα after injury. Although localizations of PPARα have been reported to occur in a variety of organs, such as liver or kidney^24^, localization of PPARα in eye has yet to be definitively clarified. Although we found that PPARα-positive cells were observed within inflammatory cells of both groups after an alkali injury, PPARα group exhibited prominent PPARα-positive cells that showed intense intranuclear staining, particularly in vascular endothelial cells, as compared to controls. A previous cell culture study that examined ratios of PPARα to γ found the ratio was 5.06 in human aortic endothelial cells, while it was 0.043 in monocytes^13^. These differences in cell-specific expression of PPARα and γ may contribute to their different roles in wound healing. In fact, PPARγ has been reported to play a role in M1 and M2 macrophage differentiation during elicitation of immune response^25–27^. Abundant localization of PPARα in vascular endothelial cells that was found in our current study suggests that PPARα may play a specific role with regard to neovascularization.

Neovascularization after corneal injury can be caused by various factors such as inflammation and hypoxia^28,29^. In our present study, PPARα agonist treatment reduced immature vascular endothelial cell proliferation during early phase, while it suppressed neovascularization during late phase. Double immunofluorescence staining also revealed that PPARα was strongly expressed in vascular endothelial cells in PPARα group, which suggests that PPARα plays a role in angiogenesis. In addition, we also found that there was a significant downregulation of mRNA expression of VEGF-A in PPARα group compared to control. Inflammation is a well-known trigger of angiogenesis, with previous reports documenting effects of anti-inflammatory agents such as NF-κB inhibitors on neovascularization^30,31^. Results of our current study confirm these previous findings. Interestingly, fenofibrate additionally decreased expression of Ang-1 and Ang-2. Furthermore, it has been reported that while blood vessel formation is regulated by VEGF along with Ang-1 and Ang-2, neither Ang-1 nor Ang-2 alone are able to promote neovascularization^32^. Asahara *et al*. have reported that addition of Ang-1 to VEGF produced an increase in macroscopically evident perfusion of corneal neovasculature, while addition of Ang-2 to VEGF assisted in initiation of neovascularization^33^. At 6 hours after injury in our current study, which is defined as an extremely early phase of angiogenesis, there was a marked increase in mRNA expression of Ang-2. However, instillation of fenofibrate significantly reduced this expression level. Moreover, during early phase neovascularization on day 4, fenofibrate treatment suppressed both Ang-1 and Ang-2. However, on day 14, which is associated with late phase neovascularization, there was no significant difference observed between these groups. Thus, these results suggest that PPARα suppressed angiogenesis by reducing expression of Ang-2 during the early phase. It has been reported that Ang-2 initiates endothelial cell proliferation by dissociating vascular endothelial cells and pericytes^34^. When considering effectiveness of therapies that target VEGF-A and Ang-2 for retinal neovascular diseases, our findings that PPARα suppressed Ang-2 in this study is intriguing from a therapeutic point of view^35^. However, since we could not definitively elucidate a relationship between these effects and the anti-inflammation effects in our current study, further investigations will need to be undertaken.

Normal cornea consists of regulated type I collagens that maintain transparency by helping to properly refract light^36^. Although type III collagens and myofibroblasts have an important role in wound healing, irrelevant deposition of collagens can result in an opaque cornea^37^, which is frequently observed after alkali burn. Our current study showed there was an accumulation of α-SMA-positive myofibroblasts and type III collagen in stromal areas. This was suppressed by fenofibrate, thereby resulting in fewer scar formations. Recent development of LV-SEM has made it possible to observe three-dimensional ultrastructural changes in tissues^38^. Therefore, we used LV-SEM for further detailed observations of these changes. Our findings revealed that arrangement of collagen fiber was smooth and regulated in fenofibrate group, while it was rough with micro-holes and corneal oedema in controls. Thus, these findings pathologically confirm the anti-scar effect of fenofibrate instillation.

In summary, instillation of fenofibrate suppressed inflammation, fibrosis formation, and neovascularization in alkali burned cornea. Results suggest that suppression of NF-kB expression is involved in an anti-inflammation effect, while downregulation of VEGF, Ang-1, and Ang-2 function is associated with an anti-neovascularization effect. Thus, PPARα agonist ophthalmic solution might be an effective treatment for severe corneal wounds associated with inflammation, neovascularization, and scar formation.

## Materials and Methods

### Animals

Eight-week-old male Wistar rats (Sankyo Laboratory Service, Tokyo, Japan) were used for all experiments in our present study (n = 10 per time point). All animal experiments were conducted in compliance with the Experimental Animal Ethics Review Committee of Nippon Medical School, Tokyo, Japan, and all procedures conformed to the Association for Research in Vision and Ophthalmic and Visual Research.

### Alkali burn model

To create a corneal alkali burn, a 3.2 mm diameter circular piece of filter paper that had been soaked in 1 N NaOH was placed on central cornea of each rat for 1 min while under general isoflurane anaesthesia. After 1 min of alkali exposure, corneas were then rinsed with 40 mL physiologic saline. All procedures were performed unilaterally in right eyes of each rat.

### Treatment with PPARα ophthalmic solution

This study used two kinds of ophthalmic solutions: a vehicle solution and a 0.05% fenofibrate solution. Ophthalmic vehicle solution was prepared using 0.1 mL polyoxyethylene sorbitan monooleate (Wako Pure Chemical Industries, Osaka, Japan) and 100 mL NaCl-based PBS (0.01 M; pH 7.4), which was prepared with disodium hydrogen phosphate dodecahydrate (232 g), sodium dihydrogen phosphate dihydrate (23.7 g), and distilled water (4000 mL). Ophthalmic solutions, including 0.05% fenofibrate, were prepared in 20 mL of vehicle solution with 10 mg fenofibrate (Wako Pure Chemical Industries). Ophthalmic solutions were kept at 4 °C. During solution administration, either 0.05% fenofibrate ophthalmic solution (PPARα group) or vehicle (vehicle/control group) was topically instilled onto ocular surfaces of each rats’ eye. Topical administration was continued in each group twice a day until reaching appropriate endpoints (at 6 hours and on days 1, 2, 4, 7, and 14 after alkali burn). At each endpoint, macroscopic photographs of each group were obtained and reviewed. Rats were euthanized at each endpoint by exsanguination under general isoflurane anaesthesia. Enucleated eyes were used for histological and immunohistochemical analyses, with real-time RT-PCR and LV-SEM performed after macroscopic examinations. For RT-PCR analyses, all corneal tissues were immediately transferred into RNAlater solution (Life Technologies, Carlsbad, CA, USA) and stored at −80 °C.

### Histological and immunohistochemical analyses

Enucleated eyes were fixed in 10% buffered formalin and embedded in paraffin for light microscopic analysis. Deparaffinized tissues were stained with HE for histopathological examination. EST staining was performed to detect infiltrating neutrophils^39^.

Primary antibodies used for immunohistochemical analysis included: monoclonal mouse anti-rat ED1 (BMA, Nagoya, Japan); monoclonal mouse anti-α-SMA (Dako, Glostrup, Denmark)^40^; polyclonal goat anti-type I collagen (Southern Biotech, Birmingham, AL, USA); polyclonal goat anti-type III collagen (Southern Biotech)^41^; polyclonal rabbit anti-PPARα (Thermo Scientific, Pierce Biotechnology, Rockford, IL, USA); monoclonal mouse anti-nestin (Nestin; Merck Millipore, Darmstadt, Germany) for detecting newly formed endothelial cells of extra- and intraembryonic blood vessels^42^; monoclonal mouse anti-JG12 (Thermo Scientific), which is specifically expressed by endothelial cells of blood vessels^43^; and polyclonal rabbit anti-NF-kB/p65 (Thermo Scientific).

For the ED1, α-SMA, type I and type III collagens, PPARα, nestin, and JG12 immunohistochemical analyses, deparaffinized tissues were stained using a standard avidin-biotin-peroxidase complex technique. Percentage of positive pixel intensity of type III collagens in 200X corneal regions was quantitatively analysed on days 4, 7, and 14 using a computer-assisted image analysis system in conjunction with colour image-analysing software (WinROOF; Mitani, Tokyo, Japan).

PPARα and JG12 were detected by examining frozen tissue sections by double immunofluorescence staining for PPARα (mouse; Texas Red) or JG12 (goat; fluorescein isothiocyanate).

### LV-SEM

After periodic acid-methenamine silver staining without a mounting cover glass, all sections were then examined using LV-SEM (Hitachi Tabletop Microscope TM3030, Hitachi High-Technologies Corp., Tokyo, Japan)^44^. LV-SEM observations in our present study compared the same corneal central area in each group. Ultrastructural alterations of corneal stroma were assessed by LV-SEM using acceleration voltages at 15 kV with 30 Pa for the backscattered electron detector.

### Real-time RT-PCR

Real-time RT-PCR was performed to investigate changes in mRNA expression of PPARα after instillation of fenofibrate. We also analysed VEGF-A, Ang-1, and Ang-2 mRNA expressions. Total RNA was extracted from corneas using Qiagen RNeasy Mini kit (Qiagen, Hilden, Germany) in accordance with manufacturer’s protocol. To ensure RNA concentration and purity (A_260_/A_280_), a NanoDrop ND-1000 V3.2.1 Spectrophotometer (NanoDrop Technologies, Wilmington, DE, USA) was used. cDNA libraries were created from 4 μg total RNA using High Capacity cDNA Reverse Transcription kit (Applied Biosystems, Foster City, CA, USA) in accordance with manufacturer’s protocol. Gene expression levels were analysed using 0.3 μL cDNA with real-time detection of accumulated fluorescence in accordance with manufacturer’s manual (ABI PRISM 7900HT, Applied Biosystems). Normalized values for mRNA expression in each sample were calculated as relative quantity of the housekeeping gene, β-actin. Primers used for real-time RT-PCR included: mβ-actin, 5′-ACC ACC ATG TAC CCA GGC ATT-3′ (forward) and 5′-CCA CAC AGA GTA CTT GCG CTC A-3′ (reverse); mPPARα, 5′-TGA ACA AAG ACG GGA TG-3′ (forward) and 5′-TCA AAC TTG GGT TCC ATG AT-3′ (reverse); mVEGF-A, 5′-TGT GCG GGC TGC TGC AAT GAT-3′ (forward) and 5′-TGT GCT GGC TTT GGT GAG GTT TGA-3′ (reverse); mAng-1, 5′-CAC CGT GAG GAT GGA AGC CTA-3′ (forward) and 5′-TTC CCA AGC CAA TAT TCA CCA GA-3′ (reverse); and mAng-2, 5′-CTT CAG GTG CTG GTG TCC A-3′ (forward) and 5′-GTC ACA GTA GGC CTT GAC CTC-3′ (reverse). SDS 2.3 software program (Applied Biosystems) was used to perform all quantifications.

### Western blot analysis

For western blotting analyses, whole cell lysates containing equal amounts of protein (25 μg) from corneal tissue were separated on 7.5% acrylamide gel by SDS-PAGE. After electrophoresis, separated proteins were transferred to polyvinylidene difluoride membranes (Invitrogen, Carlsbad, CA, USA) and incubated with anti-NF-kB (Thermo Scientific) and anti-β-actin (Sigma, St Louis, MO, USA) in order to confirm equal loading of each protein. Bound antibody was detected using appropriate HRP-conjugated second antibodies (Promega, Madison, WI, USA) for more than 60 min. Membranes were then washed and developed with SuperSignal West Femto Luminol/Enhancer solution (Thermo Scientific). Immunoreactivity on blots was detected using a LAS-4000 Luminescent Image Analyzer with CCD Camera (Fujifilm, Tokyo, Japan) and quantified, with all results expressed as ratios to β-actin protein amounts.

### Statistical analyses

All results are expressed as mean ± standard error. All values were determined by an unpaired Student’s t-test using an analytical software program (Excel, Microsoft, Redmond, WA). A value of *P* < 0.05 was considered statistically significant.

### Study approval

This experiment was approved by the Animal Care and Use Committee of Nippon Medical School (27–169).

## Electronic supplementary material

Supplementary Information
