# Supplementary material for: Peroxisome proliferator-activated receptor alpha agonist suppresses neovascularization by reducing both vascular endothelial growth factor and angiopoietin-2 in corneal alkali burn
Source: Sci Rep. 2017 Dec 19;7:17763. doi: 10.1038/s41598-017-18113-3 (PMC5736552; doi:10.1038/s41598-017-18113-3)
Supplement: Supplementary file 1 — Supplementary Information [file 41598_2017_18113_MOESM1_ESM.pdf]

Title:

Peroxisome proliferator-activated receptor alpha agonist suppresses neovascularization by reducing both vascular endothelial growth factor and angiopoietin-2 in corneal alkali burn

Names of authors;

Takeshi Arima,[1,2,*] Masaaki Uchiyama,[1,2] Yuichiro Nakano,[1,2] Shinya Nagasaka,[2] Kang Dedong,[2] Akira Shimizu,[2] and Hiroshi Takahashi[1]

Names of institution;

[1] Department of Ophthalmology, Nippon Medical School, Tokyo, Japan

[2] Department of Analytic Human Pathology, Nippon Medical School, Tokyo, Japan

*Corresponding Information;

Dr. T Arima, MD, Department of Ophthalmology, Nippon Medical School, 1-1-5 Sendagi, Bunkyo-ku, Tokyo 113-8603, Japan.

E-mail: takesuiii0714@nms.ac.jp

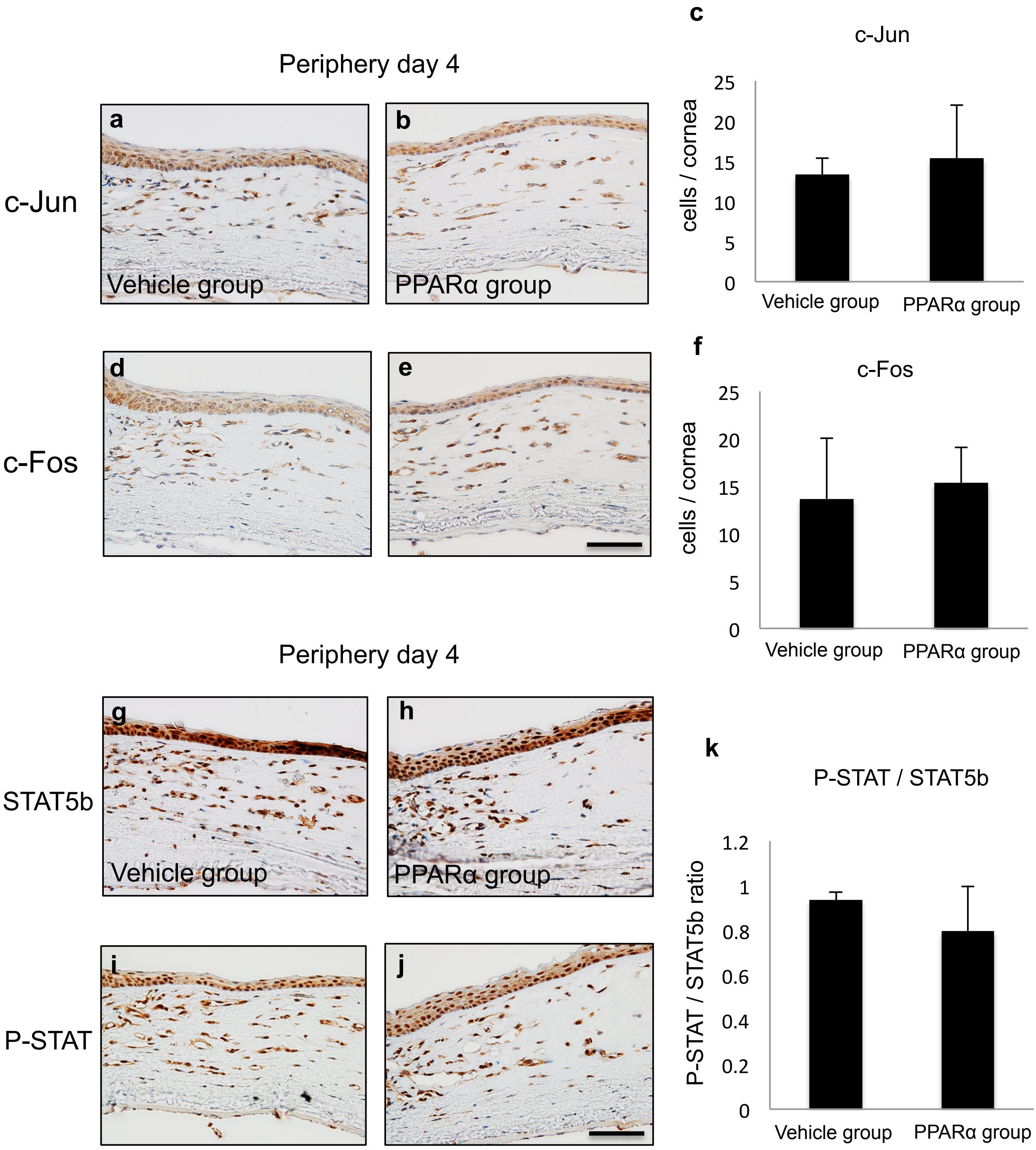

Supplementary Figure S1 Investigation of involvement of each transcription factor. (**a-f**) To examine expression levels of AP-1, immunostaining was performed using anti-c-Jun antibody (Santa Cruz Biotechnology, Santa Cruz, CA, USA) and anti-c-Fos antibody (Santa Cruz). On day 4, c-Jun-positive cells and c-Fos-positive cells were observed in peripheral stroma (**a,b,d** and **e**). Bar, 50 µm. Bar charts of number of each positive cells (**c, f**) indicates there is no significant differences between the two groups. (**g-k**) To examine activation of STAT5b, anti-STAT5b antibody (Santa Cruz) and anti-P STAT antibody (Santa Cruz) were used for immunostaining. STAT5b-positive cells and P-STAT-positive cells were observed on day 4 in peripheral stroma (**g,h,i** and **j**). Bar, 50 µm. Bar charts of ratio of STAT5b to P-STAT (**k**) indicate there is no significant difference between the two groups.
